# Moral injury research at a crossroads: assumptions, limitations, and the promise of relational reorientation

**DOI:** 10.3389/fpsyt.2026.1791225

**Published:** 2026-07-02

**Authors:** Christa Acampora, Ditte M. Munch-Jurisic, Sarah Denne, Jacob Smith

**Affiliations:** 1 Philosophy, University of Virginia, Charlottesville, VA, United States; 2 Center for Culture and the Mind, Kobenhavns Universitet, Copenhagen, Denmark; 3 Religious Studies, University of Virginia, Charlottesville, VA, United States

**Keywords:** moral injury, moral psychology, morality, relational theory, veterans

## Abstract

Our paper makes three contributions to moral injury (MI) research. First, we observe that while researchers have repeatedly acknowledged limitations with prevailing definitions of moral injury and offered alternatives, the underlying core conceptual model—which characterizes moral injury as intrapsychic damage to belief structures—has remained largely unchanged. We argue that this is a significant impediment to research progress. Second, through conceptual analysis of the most influential etiological accounts, we identify numerous implicit and untested assumptions about morality that burden the standard model. We propose that adopting a minimalist, social-functionalist conception of morality, toward which the literature is already drifting, could reduce this conceptual burden. We further hypothesize that the literature retains these assumptions because they appear logically necessary to sustain the causal link between belief violation and MI outcomes. Third, we observe that current leading treatments already target relational repair, reconditioning, and rehabilitation, which suggests that moral injury may be better understood as a form of relational disruption rather than belief violation. We acknowledge that developing this alternative would need to be pursued by the clinical research community itself. While our findings and analysis require additional development and testing to have actionable application in clinical contexts, we believe our conceptual analysis serves as sufficient justification for reconsideration of the prevailing conceptual paradigm.

## 1 Introduction

Interest in the phenomenon identified as “moral injury” (MI) has grown significantly over the past thirty years, and it is only intensifying. A robust research and clinical apparatus has grown around it. For example, the DSM-5 now acknowledges the role of moral and spiritual problems in mental health, thus providing an initial clinical framework for identifying and assessing moral injury, suggesting that “moral problems” have potential to lead to adverse outcomes (1). These developments and debates suggest it is important to provide further clarification of the concept of moral injury to improve measurement and intervention. Even leaders in the clinical research field express concern that the state of the science is “immature, characterized by the lack of a paradigmatic theory and a lack of rigor in terms of construct definition and measurement”; resulting in the inability to “generate and test hypotheses that will ultimately create knowledge about the causes and consequences of MI, and employ evidence-based assessment and intervention approaches to mitigate and treat the problem” (2). Further evidence of general agreement in the research community, across research types, that this area still lacks adequate conceptualization and definition is in the fact that researchers frequently propose new definitions to clarify or capture new case types and domain applications (2–8).

In our review of the most influential research on MI, we observe that as focus on the phenomenon has become more prevalent, researchers have acknowledged limitations with the primary definition (offering multiple alternatives) while continuing to utilize the primary core conceptual model. In previous publications, we have argued against the primary conceptual model (9). In this paper, we advance the case further through analysis of implicit and explicit assumptions of the underlying causes of moral injury, observing that the operative conceptions of ‘morality’ and the role it plays in human lives have multiple problems that potentially impede research maturation and progress. We hypothesize that this can be addressed in the research community in several ways, including adopting a minimalist conception of morality as evident in social functionalist accounts of moral injury, which could open opportunities to develop an alternative explanation to the causal relation between belief violation and MI outcomes.

Our paper makes three contributions to moral injury research. First, we observe that while researchers have repeatedly acknowledged limitations with prevailing definitions of moral injury and offered alternatives, the underlying core conceptual model—which characterizes moral injury as intrapsychic damage to belief structures—has remained largely unchanged. We continue to argue that this is a significant impediment to research progress. Second, through conceptual analysis of the most influential etiological accounts, we identify numerous implicit and untested assumptions about morality that burden the standard model. We propose that adopting a minimalist, social-functionalist conception of morality—toward which the literature is already drifting—could reduce this conceptual burden. We further hypothesize that the literature retains these assumptions because they appear logically necessary to sustain the causal link between belief violation and MI outcomes. Third, we observe that current leading treatments already target relational repair, reconditioning, and rehabilitation, which suggests that moral injury may be better understood as a form of relational disruption rather than belief violation. We acknowledge that developing this alternative would need to be pursued by the clinical research community itself. While our findings and analysis require additional development and testing to have actionable application in clinical contexts, we believe our conceptual analysis serves as sufficient justification for reconsideration of the prevailing conceptual paradigm. To use Thomas Kuhn’s terminology, we do not think that more “normal science” will facilitate maturation of this domain of research and clinical care; thus, we see this as a necessary theoretical intervention and, perhaps, provocation.

The paper proceeds as follows. Section 2 provides a literature review charting the development of what we refer to as the standard conceptual model of the etiology of moral injury. Section 3 introduces a hypothesis that the moral injury literature might benefit from application of two commonly used tools in conceptual analysis: Ockham’s Razor and a phenomenological shift taking the form of a “Copernican Revolution.” Section 4 applies Ockham’s Razor to identify the growing body of assumptions required to sustain the standard model, arguing that it is unnecessarily complex and limiting in its testability. Section 5 draws on this analysis to hypothesize alternative paradigms for conceptualizing moral injuries in terms of impacts on social, organizational, and relational contexts rather than as intrapsychic damage. Section 6 anticipates avenues for further investigation, including testable predictions, by those with the relevant clinical research expertise. Crucially, while relational factors are often acknowledged as *outcomes* or *correlates*, we argue they should also be examined as proximate mechanisms that could anchor case formulation and operational definitions.

### 1.1 Methodological approach

We approach this work as philosophers, not clinicians. Our contribution is analysis of the central conceptual morphology that is most frequently utilized in identifying and studying the phenomenon of moral injury, including its presumptive characteristics and causal mechanisms of emergence. Through critical analysis of predominant structures of argumentation in current research literature on moral injury, we identify both evident and hidden assumptions. This proceeds by identifying assumptions about moral content, causal mechanisms, etiological progression, and predicted outcomes in the most-cited theoretical articles and reviews about moral injury and from leading researchers in the field. We assess the identified assumptions for (i) parsimony (particularly the extent to which assumptions require explanatory work) and (ii) testability (i.e., whether the assumption can be operationalized in an observable way).

In this work, we draw on the literatures and methods of moral psychology, cognitive science, philosophy of science, moral phenomenology, and the broader scope of discussions of MI outside the clinical context discussed below. Following this analysis, we suggest a simplified conception of moral injury has the potential to improve testability and better explain the efficacy of current clinical treatment models, although we want to be clear that this is illustrative, not definitive—ours is not a fully-developed replacement model of moral injury but is intended as an alternative starting point from which others might develop one. Thus, although this work has implications for treatment, we do not propose new assessment tools and treatment protocols but rather suggest a framework for devising an explanatory model that is more consistent with emerging treatment plans that prioritize restoration of relational function.

## 2 Literature review of conceptual evolution

### 2.1 Early conceptions of moral injury and distress in clinical contexts

The concept of moral injury was introduced in psychiatric theory and practice through the work of Jonathan Shay (10). Shay’s lyrical examination of Homer’s *Iliad* drew comparisons between Achilles and his experience of betrayal by Agamemnon during the Trojan War and the experiences of U.S. veterans of the Vietnam War who Shay encountered in the clinic as suffering chronic mental and behavioral health problems. Shay focused on the ancient Greek notion of *themis*, roughly translated as “what’s right,” encompassing a broadly shared moral order informing expectations of proper and expected conduct. His definition of moral injury centered on the violation of *themis* by those in legitimate authority, resulting in the erosion of the moral world, the shrinkage of moral horizons, and what he described as the “undoing of character.” The underlying causes were identified as severe violations of trust caused by failures in leadership and experiences of betrayal by those with command authority and responsibility. Examples include capricious decision-making, exposure to undue risk, and failures to adequately and appropriately recognize shared grief and the burdens of responsibility and accountability. A decade earlier, Andrew Jameton introduced a similar concept of ‘moral distress’ evident among nurses who were prevented by leadership or institutional limitations and constraints from taking actions they knew to be right (11).

Shay’s original definition primarily focused on harms that were other-directed, injury done to the person by authority figures. Importantly, it was relational—rooted in betrayal of the leader-subordinate bond, a fundamental structural feature of military life—and its moral content was about the shared normative order (*themis*) rather than a personal moral code. Shay described an army as itself a “moral construction,” built and sustained through shared norms and mutual obligation and expectation. On Shay’s account, a rupture in the social order defining military organizations could result in psychological distress leading to larger and widespread dysfunction, including triggering what Shay described as a “berserker state” that could explain episodes of extreme violence and violations of expectations and perceived responsibilities.

Shay presented the concept as a way of understanding psychiatric symptoms evident in populations of veterans whose suffering was not fully captured or explained by existing PTSD frameworks. Eventually, this work succeeded in identifying a distinctive kind of harm that can be experienced by military service members, later extended to other populations, but it did not get operationalized in terms of the development of clinical instruments such as assessments and formal treatments.

Researchers examining the related concept of moral distress did, however, develop assessment measures (12), but that work largely proceeded in parallel with moral injury in military contexts and did not significantly inform it. It is noteworthy, however, that much as Shay focused on the specific institutional and organizational culture and constraints of the military to identify specific moral risks and vulnerabilities, so too did the literature on moral stress focus on the distinctive institutional and organizational culture of healthcare settings. In short, in both literatures and research streams, the sites of moral injury were anchored in relational and organizational dynamics that played a role in shaping particular moral norms and expectations. Examples of contributing factors leading to moral distress included ongoing workplace situations (understaffing, resource limitations, hierarchical barriers), breaches in professional ethics and widely accepted standards of care that are central to realizing and defining excellence in health care. Largely, evidence of harm in the examples presented were not dependent on violations of personal beliefs or individual moral codes.

**Figure 1:**
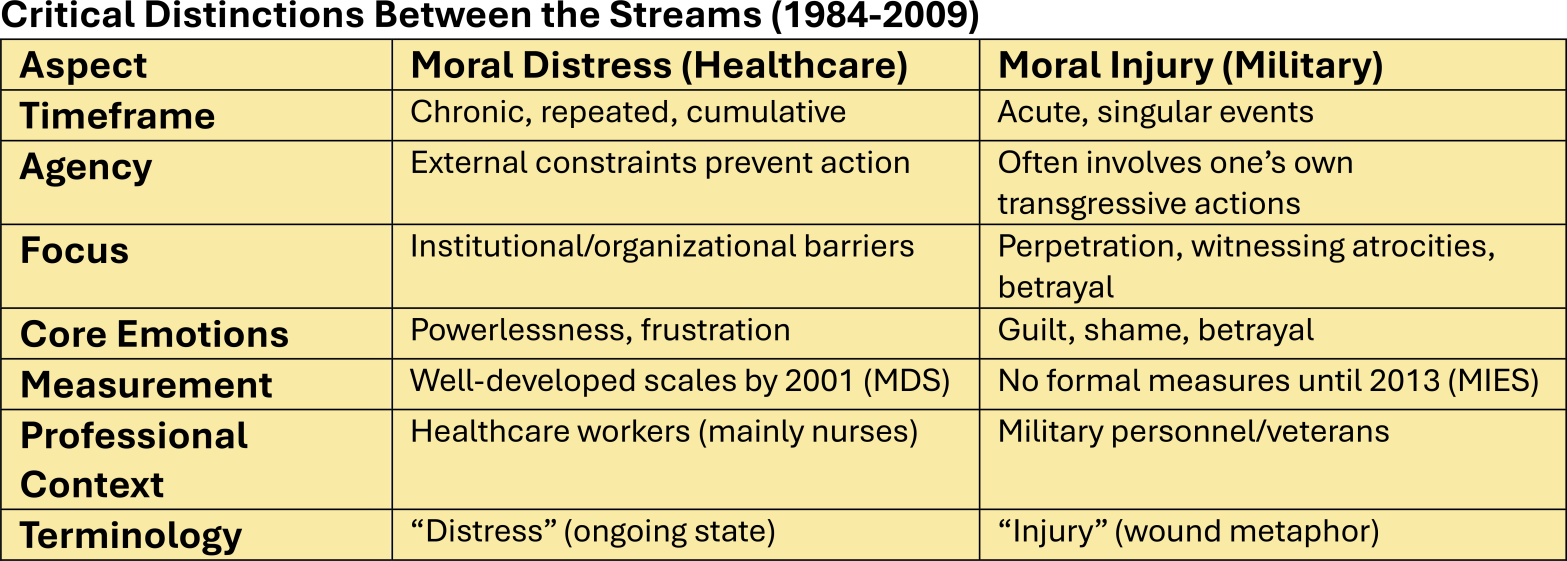
Distinctions Between Conceptions of Moral Distress and Moral Injury.

The chart below captures some of the relevant differences over the first twenty-five years of moral injury research (**Figure 1**).

### 2.2 Development of the conception of moral injury among military service members as traumatic intrapsychic damage

Following Shay’s conceptual introduction, specific dimensions of moral injury were examined to identify potential contributions to experiences of what might be regarded as *forms of moral trauma*. Specifically, potentially morally injurious events (PMIE) were examined to consider their correlation with comorbidity with but distinction from post-traumatic stress, for example researchers identified correlations suggesting killing predicted PTSD, alcohol abuse, anger, and relationship problems even after controlling for general combat exposure. What was missing was an etiological account explaining why these might occur.

In a 2009 landmark publication, a group of researchers introduced what is now widely accepted as the most prominent conception of moral injury (13). New subtypes of moral injury were introduced: in addition to the betrayal framework for potentially morally injurious events, *self-directed* actions, involving perpetrations of violence and witnessing others committing violence, were added. The study was firmly anchored in social-cognitive theories of PTSD and proposed specific intervention strategies for promoting moral repair. A significant difference between the work of Shay and Litz et al. 2009 was the new shift in emphasis from social/relational context-dependent constructions of moral expectations and forms of identification to *intrapsychic processes* and resulting conflicts. Ultimately, in that newly introduced explanatory account, the etiology of moral injury was characterized in terms of failure to cognitively reconcile moral dissonance arising from conflicts with certain moral beliefs and resulting in lingering psychological distress. Litz et al., 2009 define moral injury as “Perpetrating, failing to prevent, bearing witness to, or learning about acts that transgress deeply held moral beliefs and expectations” (13). The literature that followed developed the standard conceptual model of moral injury as an intrapsychic harm caused by exposures to high-stakes events in which moral beliefs are contravened, resulting in maladaptive beliefs and distress that manifest in a variety of clinically evident symptoms.

### 2.3 Influence and development of the intrapsychic account: the ‘standard model’

The impact of Litz et al. 2009 cannot be overstated. The Google Scholar citation count for the publication, as of this writing, is approaching 4,000, which is far greater than any other paper on moral injury. It has a Relative Citation Ratio (RCR) of 36.72, a bibliometric measure developed by the U.S. National Institutes of Health that compares an article’s citations to other papers in the same research area and publication year (14), placing it in the 99.8 percentile in terms of influence. Clearly, this is a field-defining publication, which introduced the operational definition still used today. It shifted moral injury from Shay’s seemingly narrower conception as a form of betrayal to a broader clinical construct enabling introduction of other subtypes. This created the foundation for development of various measurement instruments and treatment approaches. Importantly, this development enabled expansion beyond military contexts into healthcare (but as distinct from moral distress), first responders, law enforcement, humanitarian workers, and others.

After 2009, and accelerating dramatically a decade later during COVID-19 when healthcare workers were observed as experiencing symptoms of acute moral injury (15), the two streams—moral injury and moral stress—began to merge with the traumatic framework of moral injury and the conceptual model introduced in Litz et al. 2009 becoming dominant to the point that the terminology of moral injury and moral stress became increasingly interchangeable (16). This led to recognition that both populations have exposure to potentially morally injurious events and may face moral challenges. As a result, the current understanding, reflected in surveys of the state of the science, is that moral distress is treated as a point on a scale of intensity (somewhat less) and duration (shorter-lived) than moral injury, which is treated as the most severe and persistent. It is our view that in the historical development of conceptions of forms of “moral problems” from Shay and Jameton to what is now the “standard model of moral injury” something significant might have been lost in translation: the contribution of contextual and organizational features of moral environments in which moral injuries can occur, not primarily intrapsychic contents and structures.

As moral injury research grew after Litz et al.’s seminal 2009 article, the intrapsychic model expanded to explain how this intrapsychic damage occurs. As we explore below, this included addressing somewhat difficult questions about the origins of moral beliefs, the kinds of events that cause moral injury, and why some violations of moral beliefs cause moral injury and others do not. According to researchers advancing the intrapsychic model, people form moral beliefs through some combination of biological hardwiring and social in-grouping practices. As the theory goes, it is beneficial from an evolutionary perspective to form shared expectations about behavior with one’s social in-group. Moreover, it is assumed that much of our moral beliefs come from these shared expectations (3, 17).

Moral injury is now widely acknowledged as a “bio-psycho-social-cultural-historical-spiritual” phenomenon caused by exposure to high-stakes events that transgress one’s moral beliefs. Researchers agree that relational dysfunction and impairment are among both symptoms and outcomes of moral injury (6–8, 18–21). Recent efforts to refine the conception of MI in the clinical research literature incorporate social-functional behavioral models of sources of moral belief and formation and subsequent impacts (2, 6, 22). Nevertheless, prevailing accounts of the etiology of moral injury still ultimately characterize it in terms of intrapsychic conflict, usually in response to harmful exposure to high-stakes events, while, in the words of leading figures in the field, other people remain “reciprocal agent[s] in the background” (2, p. 268). Minimally, we argue that the circumstances and contexts of reciprocity should be brought to the foreground.

### 2.4 Concerns about the state of the science

Recently, researchers have expressed concerns that moral injury research has not produced actionable data on how to conceptualize moral injury and how to prevent it or treat it (2, 3, 23). Even leaders in the field observe that studies on moral injury widely suffer from concerns about content validity—they do not reliably show a relationship between certain causes and morally injurious outcomes in alignment with accepted scientific practice (3). Litz expresses concern that the scales used for assessing moral injury may be unreliable tools, in part due to their reliance on a patient’s own assessment of the event(s) in question and the outcomes of it (3). Litz proposes that including more social-functional considerations in moral injury studies will allow for more reliable empirical studies on the phenomenon. Ultimately, we agree with this, and we make suggestions for how social-functional considerations might become more prominent.

## 3 Hypothesis

Our view is that what has become the standard conceptual model of moral injury requires an overly complex explanatory apparatus to account for the sources, nature, and functions of moral beliefs and experience (9). This results in misidentification of the proximate causes of moral injury as violations of persons’ moral belief structures. Below, we provide examples of two sets of assumptions that are implicitly or explicitly evident in the literature. These include assumptions about the origins of moral beliefs and the etiological characterization of moral injury as damage to those beliefs. We argue that rather than clarifying how and why damages to moral belief structures result in adverse outcomes, these assumptions impede empirical testing and are ultimately unnecessary for research progress. To advance moral injury research and support devising more adequate conceptualizations, we recommend application of two philosophical tools: “Ockham’s Razor” and a phenomenological shift taking the form of a “Copernican Revolution.” To be clear, this does not result in a specific replacement conceptual model, but it does suggest potentially promising avenues for other clinicians and researchers to explore and develop. Importantly, it also suggests that more caution might be warranted in the promulgation of the standard model and its associated instruments, which are now undergoing processes of review and translation into multiple languages (24, 25).

### 3.1 Ockham’s Razor

Ockham’s Razor, sometimes called the Principle of Parsimony, is attributed to the 14th-century philosopher William of Ockham. It posits that when faced with competing hypotheses, the simplest, namely, the one that requires the fewest assumptions, is preferable. In medical research, application of Ockham’s Razor can guide favoring simpler explanations over those that are more complex, provided the simpler sufficiently accounts for the observed data and supports development of effective interventions. Applying this tool can help reduce diagnostic errors and unnecessary interventions by prioritizing the most straightforward evidence-based conclusions.

### 3.2 Copernican Revolution

A “Copernican Revolution” entails a paradigm shift that radically alters how a field or area of study conceptualizes its primary phenomena and foundational principles. This supports novel perspectives that can lead to new discoveries, much as Copernicus’ heliocentric model overturned geocentric cosmology. To be clear, such a transformation of thought is not derived solely from incremental insights; rather, it creates a new framework that enables new kinds of inquiry and discovery.^1^ Applied in this context, a Copernican Revolution might not produce a radically new model of moral injury, but could re-center the cause of moral injury from intrapsychic beliefs to moral relations in various social contexts.

Application of these two tools, especially in tandem, could lead to more robust accounts of the mechanisms of moral injury, resulting in improved diagnostic instruments, more targeted and effective treatments, and advances in prevention. In short, we hypothesize that the application of Ockham’s Razor can result in a simplified conception of moral injury that can also support a paradigm shift in examining the causes of injury from focusing on intrapsychic moral belief structures to examining the world of interpersonal relationships most relevant for moral functioning.

## 4 Applying Ockham’s Razor

Current clinical research on moral injury advances unnecessarily complex and untestable views relating to moral belief formation and systematic maintenance of those beliefs. These include implicit assumptions that are necessary to sustain the now-standard model of moral injury that construes it as damage to intrapsychic moral belief structures (9). Following Ockham’s Razor, when assumptions are unnecessarily complex or unjustified, they can be jettisoned for a simpler theory that advances research and clinical activity.

### 4.1 Primary assumptions about moral phenomena and function

All theories require at least some assumptions whose unfounded and untested dimensions are then reduced and clarified through research and investigatory processes. Moral injury research, however, appears to be developing in the opposite direction, increasingly burdening the conceptual framework with additional assumptions. We believe this impedes understanding and treatment of moral injury. A fulsome review of these assumptions would require a lengthier account in a different venue, so we limit our survey to those that are most common and discuss several salient examples to illustrate the larger point.

A primary assumption in moral injury research is that moral injuries arise from harmful transgressions of one’s moral beliefs, whatever forms those might take. Leading hypotheses make multiple additional assumptions, typically including:

1. The core basis of morality is moral beliefs of various kinds (linked with values, norms, rules, codes, principles, virtues, etc.; the literature equivocates in using such terms) (13, 30–32).
2. On a phylogenetic level, moral beliefs have various origins: some are hardwired evolutionary biological processes (particularly, group identification and preference) (2); some reflect various social, cultural, religious, and spiritual formative inputs (6, 33, 34).
3. Despite their various origins and forms, moral beliefs are generally grounded in expectations of reciprocal altruism from members of one’s social in-group (2).
4. On an ontogenetic level, the development of moral beliefs largely tracks human bio-social development (3, 6, 23).
5. At a maturation point, moral belief structures and contents stabilize and become fixed (e.g., a “healthy” or intact “conscience” (4, 35); perhaps even rigid in some persons (36)).
6. Exposure to certain kinds of high-stakes, non-distal events can threaten moral beliefs. These are specifically identified as Potentially Morally Injurious Events (PMIEs) (13, 19, 21, 37).
7. In at least some people, when moral beliefs are contravened in contexts of exposure to a specific PMIE, this can result in traumatic damage to the belief structure itself such that it has pervasive impacts on social functioning (3, 38).
8. Trauma to moral belief structures is a form of stress (on a continuum of potentially injurious experiences) that can result in psychological injury resembling post-traumatic stress disorder (13, 17, 19, 39).

### 4.2 Primary framework assumption: “events” can cause traumatic damage to moral belief structures

One of the key assertions of the current conception of MI is that “events” are primary causes of moral injuries. This claim imports a vast array of assumptions at the root of clinical practice and research that are reified in relevant instruments such as the Potentially Morally Injurious Events Scale (PMIES), the Moral Injury Events Scale (MIES), the Moral Injury and Distress Scale (MIDS), and the Moral Injury Questionnaire (MIQ) (40). This has resulted in approaches to case construction and treatment that primarily follow protocols and research methodologies arising from and drawing on literature in trauma studies, possibly because the identification and study of MI arose in contexts of treating U.S. Veterans presenting with symptoms of post-traumatic stress. This entire conceptual framework relates to a cluster of assumptions that should be considered candidates for critical analysis and application of Ockham’s Razor.

It is unclear, and we believe untestable, whether beliefs or belief structures themselves can be subject to traumatic injury (much less, if so, whether this can be causally linked with an event). Consequently, it is also unclear whether treatment models for other forms of trauma are more or less likely to be effective for promoting repair to those belief structures. Furthermore, there is evidence in numerous first-person accounts that moral injuries are not necessarily (or exclusively) connected with particular events that in themselves cause damages to belief structures. For example, recent studies show that many Veterans identify certain phenomena as morally injurious that are not readily classifiable as discrete events, such as military financial waste and diffuse social phenomena such as sexism and racism (41). These experiences are not typically connected only with specific events but rather patterns of social behavior, including lack of appropriate concern for responsibilities and relational expectations. Another example is evident in Tyler Boudreau’s account of his experience as a Marine deployed in Iraq in 2004. Although he acknowledges his own moral injury, he does not identify any specific high-stakes events as the cause. Instead, he links his moral injury with longer-term experiences of soldiering and the structure of his relations and interactions with civilians in conditions of occupation (42).

Experiences of MI that do not fit the ‘event model’ are also evident in accounts of MI in healthcare contexts in which structural conditions prevent adequately delivering care (43) as well as other workplace examples (44) and in cases of experiences with displacement (34, 45, 46). Examples of a broader range of experiences linked with MI that seem to not fit the trauma framework suggest that the trauma framework might not be the most relevant. While those asserting the aptness of the event model might argue that such examples are not, in fact, instances of moral injury, a greater portion of the burden of proof would seem to lie with them, since there are studied instances of presentations of the same symptoms of moral injury in terms of severity and impairment in these cases.

Moreover, the trauma framework could be limiting in other ways, since it is likely that the assumption that MI must be a form of trauma also inclines researchers to overlook other considerations such as relationships and relational dysfunction or degradation, which we hypothesize are more significant and relevant than belief structure damage when assessing and addressing MI. In short, the limitations of the assumptions of the trauma framework potentially create a blind spot that might otherwise afford differing etiological accounts, features salient for case construction, and more efficacious treatment modalities.

### 4.3 Secondary assumptions about moral phenomena and function

Beyond the attention to events, the primary assumptions have resulted in a cascade of other assumptions. Assumptions about the complex origins of moral beliefs also influence how researchers think about the general (normal, typical, healthy) contents of moral beliefs and their durability. For example, some researchers ascribe as typical a view of the world in terms of evident goodness and badness (or evil), drawing on metaphysical assumptions that are potentially incompatible with various cultural and religious traditions (47).

In addition to vague assumptions about the nature, organization, and function of moral belief structures, the literature lacks precision in discussion of various kinds of moral phenomena, conflating and often lumping together without distinction moral phenomena of very different kinds. For example, researchers in this area often equivocate on norms, rules, codes, beliefs, virtues, and even behavioral repertoires (3, 9) overlooking the wealth of literature emerging over millennia articulating how these phenomena are distinct (48–54).

Moreover, routinely and in everyday experience, we witness others violate our moral beliefs and that others hold dissonant moral beliefs—this occurs without experiencing moral injury. The very fact that moral beliefs are typically quite fluid and flexible, requiring constant negotiation and renegotiation should lead us to be suspicious about the conceptual construction of moral injury as occurrent in events that break fixed moral belief structures (52, 54, 55). All these assumptions—the primary and explicit, as well as those that are implicit—are subject to considerable contestation and qualification. Importantly, most are not amenable to the type of hypothesis-testing that is necessary for grounding empirical clinical science.

### 4.4 Selective accounts and assumptions of origins of primary moral beliefs and structures

There is a general trend in MI clinical literature to selectively adopt and prioritize propositions supporting the view that the most important moral ideas and tendencies human beings have are rooted in evolutionary biological development. Consequently, there is little if any consideration of the enactive and interactive dimensions of bio-socio-cultural entwinement that has been acknowledged in operative definitions of moral injury but not reflected in accounts of moral functioning (54, 56–59). While the clinical literature generally acknowledges that moral beliefs and notions are strongly rooted in bio-psycho-social-cultural-historical-spiritual development, very little of the social-cultural-historical *particularities* and *contexts* of moral beliefs are considered and explored in the primary MI literature. Much of it bluntly construes morality in terms of a structure of “Us-group rules” (3, 47) whose abiding is somehow linked with overarching self-assessments of moral worth, estimations of value by others, and overall “life affirmation” (13).

Some exceptions to this tendency, reflecting also divisions in treatment approaches, are those views of moral injury that also try to account for spiritual and religious bases for origins of moral belief (8). However, even in those instances, confusion about the nature of differing moral phenomena persists as does the fundamental conception of the etiology of moral injury in terms of harm to intrapsychic structures. It is worth noting also that the existence of “Us-group rules” is neither established science nor more amenable to empirical observation and testing.

Our view is that this narrowing of focus occludes understanding of the relational contexts in which moral functioning occurs in both normal and abnormal circumstances. One solution might be to expand the explanatory apparatus to attempt to include these features, adding further to the complexity of the assumptions identified above. We think a different approach is also possible. As we discuss below, a Copernican Revolution for MI research could help in achieving a wider, more encompassing perspective.

## 5 From mind to world—a Copernican shift

The application of Ockham’s Razor reveals unfounded assumptions required to sustain the view that moral injury arises as an episodic intrapsychic stress injury. To advance the science and treatment of MI, a new paradigmatic orientation, not just a new definition, is needed. One possibility is to replace the focal point on moral beliefs and their violation (centered in the psyche) and instead bring to the foreground the idea that morality in practice is something that takes place between and among people (within a social universe). There are many arguments for this view outside the MI literature (52, 54, 60–62) and within the MI literature (9, 55, 63). Clearly, moral beliefs are relevant to our moral encounters in the world and our assessments of them, but morality is much more than just whatever moral ideas or beliefs we might have at any particular time (64). Shifting the focus of moral relevance to interpersonal relations and practices in the world would constitute a “Copernican Revolution.” This reoriented perspective could serve as the basis for a hypothesis that moral injuries arise from damages to moral relations, not necessarily primarily in damages to moral beliefs (9).

### 5.1 A minimalist, social-functionalist view of moral practice

What exactly morality is and where it comes from are topics of debate about which there are many competing and complex views. However, for the purposes of conceptualizing moral injury, we think it is enough to begin with a primary observation that moral encounters occur *in the world* and not strictly in contexts of “event exposures” and our reflections on them. Whatever the sources of belief might be and whatever ways persons typically engage in moral decision-making as motivation for action or evaluation of the actions of others, the practice of morality takes place in the world and is contingent upon and continually shaped and constituted by our interactions and negotiations with others (52). This happens in a great variety of interactions and contexts of forms of relationship and relatedness (e.g., with other individual and corporate actors, non-human beings, religious and spiritual entities, features of our environments, etc.). From this viewpoint, it is plausible to consider that moral injuries can be viewed as damages to our capacities for moral relating, connected with specifiable contexts of relationships, and not primarily as damages to our belief structures.

To be clear, we think of this as a minimalist conception, one that provides a sufficient basis for accounting for forms of moral harms people can experience. It does not encompass everything we might want to know about formation of beliefs or forms of moral reasoning and how these do (or do not) relate to action. The focus on observable moral relations as evident in human interactions rather than contents of beliefs and intrapsychic structures is largely theoretically neutral—any moral theory that is amenable to the relational and practical character of morality would be consistent with our proposed Copernican shift. From a moral philosophical perspective, this could include strands of virtue ethics (65–67), communitarian ethics (68, 69), feminist ethics (52, 54, 70), constructivist ethics (71–73), phenomenological ethics (74, 75), as well as many others. It is enough for the purposes of this area of clinical research, we think, to stick to the observable relations and interactions in the world to find evidence of moral salience in people’s lives. This view has advantages in terms of potential application in broader and cross-cultural settings.

There is a wealth of literature on the nature and development of moral experience that researchers might consider. Here we are simply arguing for the distinction between experiences (of not only discrete and specific events) and the contexts in which they occur in relationships of various kinds, including with oneself (not only through engagement with or as impacts on specific beliefs or belief structures). In this way, the specific conditions of relatedness that are disrupted in cases of moral injury could be a primary target of observation when assessing for moral injury and developing case construction, and we think the results could lead to diagnostic and therapeutic breakthroughs. Consideration of the example of the concept ‘respect’ can help illustrate this point. It is a particularly apt example because it is important in both major strands of the MI literature (i.e., moral distress and moral injury) and crosses the subtypes identified in the clinical constructions of what we call the standard model. There is a rich philosophical literature examining respect as a moral phenomenon, spanning its differing forms, contents, relational structures, and functions in moral life, from Darwall’s foundational distinction between recognition respect and appraisal respect (76), through Dillon’s work on care respect (77), Margalit’s analysis of respect as a property of social structures (78), and Hill’s investigations of self-respect and autonomy (79). Taken together, this shows how respect is not merely a single norm or code provision but a complex, context-dependent moral phenomenon operating across multiple registers (71, 80).

### 5.2 ‘Respect’ as illustrative example

Respect can take many forms, is relative to its context, and acquires its meanings in different domains in relation to specific actions people take. Moral contents related to ‘respect’ are not reducible to any single moral-theoretical category (norm, rule, virtue, code provision); its various meanings are constituted by specific actions and interactions in specific relational domains and shift over time as those practices change; moreover, this character of moral phenomena supports empirical research that is more tractable because it is more evident in observable behaviors than research premised on intrapsychic moral belief structures.

When ‘respect’ functions as a moral rule, as in “Respect your elders,” it prescribes a class of behaviors toward a class of persons. Even as a rule, however, its content is context dependent. What counts as respecting one’s elders differs not only across cultures but across specific relationships: deference to a grandparent at a family gathering, yielding the floor to a senior colleague in a meeting, physical gestures of acknowledgment toward elders in certain cultural contexts. While the rule may be stable, the actions that satisfy it are not specifiable without knowing the relational context.

When ‘respect’ refers to a legal obligation, as in “Respect the speed limit,” it functions as an expectation for compliance, disclosing an ambiguity between beliefs and practices. One can comply without respecting the law itself (grudging obedience), and one can respect the rule of law while deliberately violating a specific statute (civil disobedience). Even in the legal domain, respect names a relationship to authority that can take differing behavioral forms.

Notions of ‘respect’ can transcend multiple contexts, as in a general disposition to “respect life” itself, evident in various religious and spiritual traditions. When taken in this way, “Respect life” is a source of many tensions that the abstract imperative might imply. Such tensions could include situations involving euthanasia or abortion, which reveal tensions between whose life is respected and the specific forms that genuine respect for life might require.

Expectations and conditions for ‘respect’ are also common in professional ethics. The American Nurses Association Code of Ethics’ opening provision is instructive because it does not treat respect as a standalone rule (81). Respect appears alongside compassion and is directed toward “inherent dignity, worth, and unique attributes of every person.” It functions as a practical orientation enacted in concrete clinical encounters, how a nurse speaks to a patient, handles a body, communicates a prognosis, or navigates a disagreement with a family member about care. A nurse who follows every procedural rule but treats patients with indifference would violate the code, because what the code demands is not rule-compliance but a particular quality of relational engagement.

‘Respect’ also plays a significant role in military contexts, as illustrated in Shay’s formulation of moral injury as a violation of what is right both in a warrior tradition as well as the broader society. In a wide variety of traditions of military ethics, certain extreme actions are permissible only on the condition that other forms of respect remain intact: respect for the enemy as a combatant and fellow human being, respect for the dead, respect for non-combatants. In these cases, ‘respect’ is not one rule among others but the moral condition that makes the entire framework of permissible action coherent. When respect collapses (e.g., when enemies are dehumanized, when the dead are desecrated, when non-combatants are treated as expendable), the overarching permissibility structure itself collapses, and what was previously understood as duty can become connected with excessive abuse, atrocity. Examples in the literature suggest that injury often occurs precisely at the point where the conditions of respect that make actions and relations morally sustainable are violated or revealed to have been absent.

These examples show how ‘respect’ appears in differing contexts or domains and is instantiated in differing relational formations, including in: embodied practices such as handshakes, bows, and salutes; informal interactions of holding each other to account through scolding and praising; various forms of restraint, as when avoiding prying questions when someone appears distressed; and in civic contexts, as a political practice in which forms of speech and persuasion advance or impede conditions for shared life in common.

The cumulative force of these examples is that respect cannot be located in any single code, norm, value, or rule. Instead, it functions as a condition for the possibility of moral relationship and acquires its specificity only in relation to particular persons, contexts, and practices in which it is enacted. An intrapsychic belief-structure model has difficulty capturing this kind of moral phenomenon, because the “belief” that one should be respectful is almost empty without the relational context that gives it content. The meanings of respect are not simply inherent in beliefs prior to and then expressed through actions: those meanings are constituted by the actions and interactions themselves, and they change as those actions change. Any abstract expression of a belief about the importance of respect is a retrospective gloss on what is a dense, shifting, historically particular set of practices. Importantly, these are specifiable when subject to analysis, and they are observable as present or absent in particular cases.

This suggests that when moral injuries occur relating to violations of respect, what is damaged is not an abstract belief that one should be respectful, owed respect, or that the world is just. What is damaged is the capacity to participate in the specific practices of respect, understood as the specific relational actions that give moral life its texture and meaning in a particular domain. This could explain why persons with moral injuries report experiences such as a loss of a world, not just a loss of a belief: the practices that constituted one’s moral world have been rendered unintelligible, impossible, or hollow. This also suggests why recovery cannot be achieved simply by restoring or revising a belief or belief structure. If the meaning of respect is constituted by practices and interactions, then repair must seek to reconstitute those practices and interactions, by new or restored forms of moral relating that gradually reconstitute what respectful engagement means for this person, in this context, given what has happened. That is a relational and practical undertaking, not a cognitive one, which has therapeutic implications.

Much of the moral injury literature implicitly assumes a stable and integrated moral belief system such that when one element is violated, the whole structure can be at risk. That assumption does significant work in the standard model—it is what makes it plausible to talk about “shattering” moral beliefs, about the collapse of global self-assessments of moral worth. But if we take seriously what the respect examples show, there is no special reason to expect that a person’s moral life is organized as a unified system. A person’s moral engagements in their family, professional role, civic life, friendships, and faith community are not necessarily integrated into a single coherent framework. They may coexist in considerable tension. This is not necessarily moral confusion or dysfunction; it is a normal feature of moral life as it is actually lived. People inhabit multiple domains of moral practice simultaneously, and those domains have their own internal logics and evolving standards.

This distinction matters therapeutically. If the injury is to a belief system, then the therapeutic task is to repair or reconstruct the system. If the injury is to specific moral relationships and practices, then the task is different: to identify which relationships and relational capacities have been damaged, to understand the specific relational context in which the damage occurred, and to work toward the restoration or reconstitution of moral practice in that domain. This may involve entirely new relationships and new forms of practice, not simply revised beliefs about old ones.

The relational orientation that could be realized through the Copernican Revolution we suggest does not merely offer a different theoretical account; it redirects empirical attention toward phenomena that are, by their nature, more amenable to observation, specification, and measurement. Our hypothesis is that an alternative framework of this kind might support the development of stronger empirical research. If moral beliefs and meanings are realized, given their specific contexts and meanings in persons’ lives, in and through experiences and interactions with others, then these are observable and specifiable. Because the domains are specific, it is also possible to be concrete about the ways in which the beliefs and meanings are or are not evident, in specific interactions among specific individuals as well as among groups or even organizational structures.

### 5.3 The primacy of moral relatedness and relational context

Contemporary MI research that advances the standard model includes sensitivity to the social-functional contexts of belief formation and relational impact, which we regard as a positive development (2, 33, 47), but we argue that it does not go far enough. When devastating moral experiences transpire, whether episodic or cumulative, the relational context and the specific relations that are ruptured are at least as important as the “event exposure.” Recent lines of research emphasize the collapse of moral worlds, primarily through relatedness to others, as among the key features of moral injury. Litz and Walker write, “MI, at its core, is about the loss of kinship (i.e., belonging and being part of something meaningful), pride in self and others, caring and trusting relationships at home, at work, and in communities” (2, p. 267). Moreover, recent characterizations emphasize the primacy of social pain and “a loss of rewarding, comforting, and safe relationships” (2, p. 265). Sources of belonging arise from contexts (home, work, and community) that are rooted in relationship with others, not among the contents or integrity of beliefs or belief systems.

Despite this, current models do not foreground the relevant relational context. Instead, they acknowledge the others connected with the “event” precipitating MI as figures who are “reciprocal agent[s] in the background” (2). Our claim is that these agents are not simply “in the background,” and the relationship itself (and its rupture) should become a predominant object of attention. From our point of view, it is necessary to understand how and why the rupture of the particular form of relatedness was so important to the person who experiences MI and how it went awry. “Reciprocal agents” are never generic; rather, they are always specific persons or groups of persons with whom one is in relation.^2^ These relations should be placed in the foreground. They are specific and proximate to the injury. Moreover, as we discuss below, we think understanding these relations is also likely indicative of more specific restorative pathways.

If moral injury involves the disruption of specific moral relationships and the specific practices of moral engagement within identifiable domains, then the primary objects of inquiry are interactions, behaviors, and relational patterns—things that can be observed, described, and in many cases corroborated by multiple sources. One can ask not only the injured person but also the people with whom they are in relationship. One can observe changes in specific relational behaviors over time. One can identify the particular relational domain in which the disruption occurred and specify what the practices of moral engagement in that domain looked like before, during, and after the injurious experience. This opens several concrete research possibilities that the standard model does not readily support.

**Figure 2:**
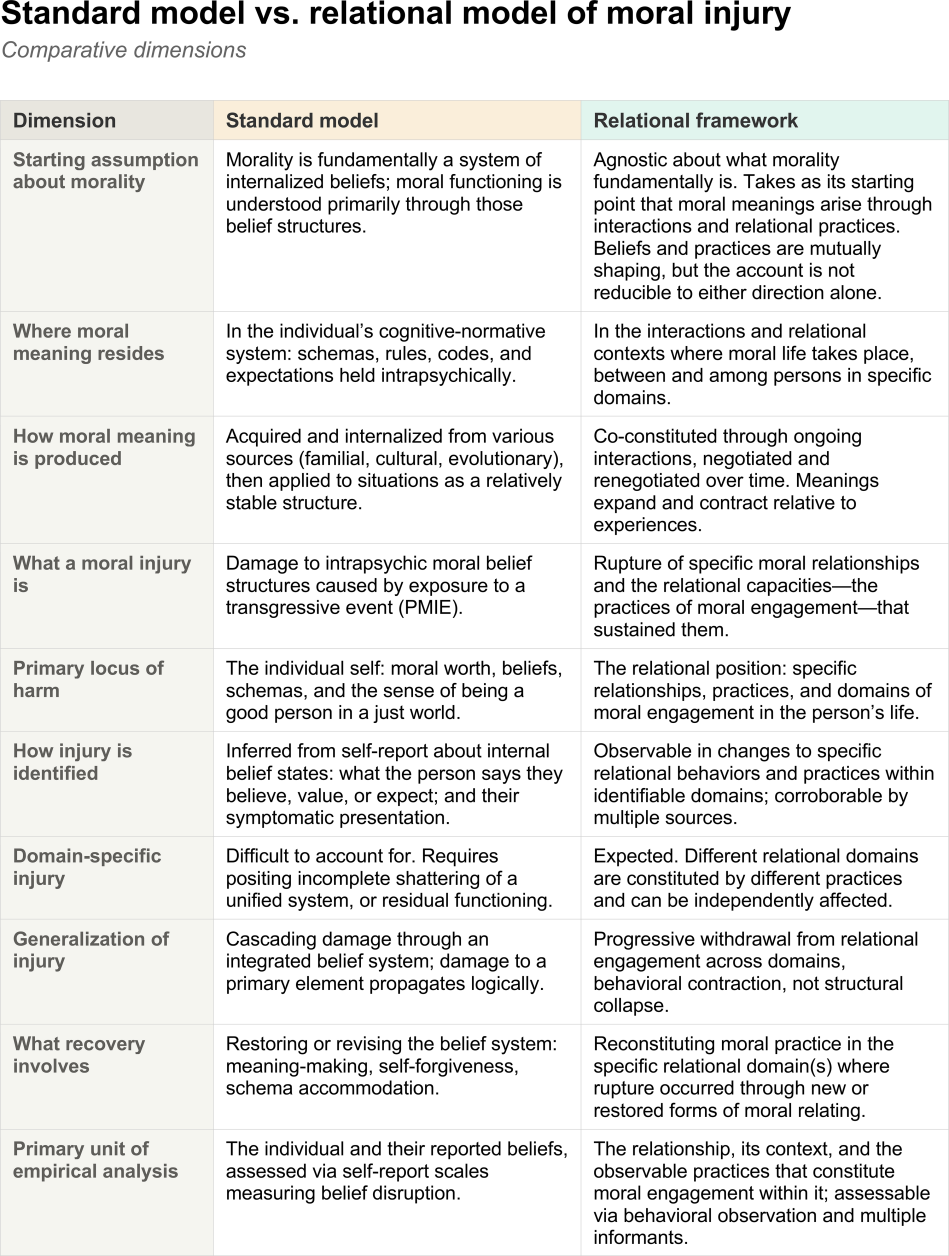
Comparison of Standard and Relational Models.

Consider the asymmetry: under the standard model, the core object of inquiry is a moral belief structure that is internal to the individual. To study it, researchers must infer its contents and organization from self-report. This is not just a practical inconvenience; it introduces a fundamental methodological problem. Self-reporting about moral beliefs is notoriously unreliable, not because people are dishonest but because moral beliefs as the standard model conceives them—deep, tacit, organizing structures—are precisely the kind of thing people may not have reflective access to. A person may not be able to articulate what their moral belief structure contained before the injury, what specifically was violated, or how the structure has been altered. The measurement instruments that result—scales asking people to endorse statements about their moral beliefs—are measuring something, but it is far from clear that what they measure corresponds to the theoretical construct they are designed to capture. The table below captures salient points of comparison and distinction between the standard model and the relational framework (**Figure 2**).

## 6 Promise of the new framework

As the “state of the science” now stands, there is a laundry list of potentially efficacious therapeutic approaches that have shown some clinical promise (2, 47); however, there is not yet an established explanatory basis for why some of these lead to recovery and only in certain cases, and why others do not. Presently, the clinical literature has little firm established basis to distinguish which therapeutic pathway is more likely to be effective for not only reducing symptoms but ultimately restoring moral function, potentially resulting in treatment plans that are too generalized and not specific.

### 6.1 Clinical translation

The paradigm shift we propose potentially facilitates advances in precision across five dimensions of clinical research and practice: identification, measurement, longitudinal observation, case construction, and targeted treatment. In each of these areas, we suggest that the relational framework could result in greater explanatory power with greater parsimony, generate testable predictions, and promote overall wellness rather than primarily reduction in symptoms. We further conjecture, although this is outside of our domain of expertise, that the larger fields of psychology and psychiatry possess relevant tools and frameworks that could be usefully applied and adapted for understanding relational function in a variety of domains of human organization, so that adoption of the relational framework does not require that moral injury research must begin again. In what follows, we outline the promise each dimension holds and, where possible, offer specific testable predictions that the relational framework generates and that the standard model does not.

### 6.2 Potential for greater precision in identification

In the standard model, the context is essentially determined by an event type taxonomy. The relational framework allows researchers to identify not just what happened but the specific relational domain and structure in which it happened, to more clearly consider what the moral expectations of the particular relational domain were, how they were constituted in practice, what specific relationships were involved, and how the injurious experience disrupted those specific relational configurations. Thus, two people exposed to superficially similar events (e.g., for example, two health professionals who both experienced institutional failures during a pandemic) could be understood as having experienced quite different moral injuries if their specific relational contexts differed. One might have experienced the rupture of a relationship with a specific mentor or team; another might have experienced the collapse of a relationship with the institution as a moral entity. Injuries arising in these different contexts could have different phenomenologies, different consequences, and potentially require different recovery pathways, because they occurred within different social/relational spaces. These are distinctions that an event-type taxonomy cannot capture but that a relational assessment potentially could.

This suggests that types of moral relationship might be more significant than event types. Tools for assessing this already exist, such as the Meta-Cognition Assessment Interview and the Meta-Cognition Assessment Scale that measure relational dynamics, including perceptions of self and others (82). Application or adaptation of these assessments could also include criteria for what counts as moral relatedness (rather than codes, norms, values, and beliefs), drawing more broadly on the well of literature that explores this including: accountability and answerability, obligations arising from professional and social roles, various forms of mutual recognition, expectations for trust and loyalty, and norms emanating from community membership. As just one example, a recent study of moral experiences, risks, and potential injuries among “peer support specialists” who serve in alternative response programs explicitly drew on complaints within the community in the call logs and field reports as one data source among numerous others to understand the concrete and specific norms and expectations that arise from the socio-cultural context in which the responders serve (83).

If the relational framework is correct, then among individuals exposed to superficially similar potentially morally injurious events, the severity and character of moral injury will be better predicted by the type of moral relationship disrupted—such as peer-level mutual accountability, hierarchical trust, or institutional belonging—than by event type alone. This could be tested by comparing the explanatory power of relational variables against event-type classifications in predicting functional outcomes across a sample of individuals who experienced similar exposures in different relational configurations.

### 6.3 Measurement

The domain-specificity of the relational framework potentially allows for more precise measurement of both injury and recovery. If we know that moral engagement in a particular professional context involves identifiable relational practices, then we can develop instruments that assess the functioning within those specific practices rather than inquiring about global moral beliefs and their origins. Moreover, examining the examples of moral injury in military, healthcare, and other contexts suggests that these relational practices *can* be specified with considerable precision. Relevant assessment terms could include: Are the specific interactions that constitute respectful engagement in this domain still occurring? Have they changed in identifiable ways? Are new practices emerging that serve similar relational functions? These are questions that can be answered through behavioral observation, structured interviews with multiple informants, and even organizational data, not only through individual self-reporting.

If moral injury manifests primarily in disruptions to specific relational practices, then instruments assessing changes in identifiable relational behaviors within specific domains, such as frequency and quality of professional consultation, patterns of mutual accountability, or participation in communal moral practices, should demonstrate stronger predictive validity for functional impairment than instruments assessing global moral belief disruption. Moreover, multi-informant assessments that incorporate reports from others within the affected relational domain could yield higher inter-rater reliability than self-report measures of internal belief states, because relational behaviors are accessible to observation by multiple parties.

### 6.4 Potential for longitudinal study

The relational framework supports longitudinal research in ways the standard model does not. Because moral practices and relational patterns are observable over time, researchers can study how moral engagement in a specific domain evolves before, during, and after potentially injurious experiences. This is important because, as we and others argue (9, 41), moral injury need not be episodic. It can be cumulative, unfolding over time through the progressive erosion of relational practices. A longitudinal relational approach could track that erosion as it occurs rather than relying on retrospective accounts of singular shattering events. It could also track recovery as the gradual reconstitution of moral practice in specific relational domains, rather than as the revision of a belief structure at a single point in time or the reduction of symptoms.

If moral injury unfolds through the progressive erosion of relational practices rather than through a particular shattering of a belief structure, then prospective longitudinal tracking of specific relational behaviors—such as patterns of engagement with peers, leaders, or institutions—should detect measurable changes in moral practice prior to the onset of the symptomatic presentations typically associated with moral injury. Similarly, recovery trajectories should be identifiable as the gradual reconstitution of relational practice in specific domains, rather than as a discrete moment of belief revision, and should be trackable through behavioral indicators accessible to multiple observers.

### 6.5 Case construction

Case construction potentially becomes more specific and more anchored in observable phenomena in the relational framework. Rather than assessing whether a person’s global moral beliefs have been violated, which requires the person to report on internal states that may be opaque even to themselves, a clinician or researcher can map specific relational domain(s) in which the injury occurred, identify the specific relationships that were disrupted, and describe the specific practices of moral engagement that have been lost, distorted, or rendered impossible. This is potentially empirically richer because it deals in observable particulars rather than inferred generalities (84). A clinician could document, for instance, that a Veteran who previously maintained specific practices of mutual accountability with fellow service members has ceased those practices following a betrayal by leadership, for example. The practices tracked by the clinician could include checking in after missions, sharing responsibility for difficult decisions, maintaining physical proximity and verbal honesty in debriefing. The injury is more evident in the relational behavior than in self-reported beliefs alone (83).

### 6.6 Potential for targeted treatment

Finally, if recovery pathways are indexed to the type of relationship, then treatment outcome research can be designed with greater precision. Rather than asking whether a treatment reduces global moral injury scores on a self-report scale, researchers can examine whether it restores specific relational capacities in the specific domain where injury occurred. This allows for the testable prediction that different therapeutic approaches may be differentially effective for various relational configurations of moral injury, a hypothesis that the standard model, with its unified belief-structure orientation, does not generate but that the relational orientation makes testable.

In this case, for example, one might test the prediction that interventions emphasizing relational repair (e.g., community reintegration, trust rebuilding, adaptive disclosure/acknowledgement) will outperform cognitive “belief restructuring” in cases where betrayal/kinship loss is primary.^3^ If damaged relations are targeted for discovery in identification of MI and case construction, this could facilitate understanding precisely which relation or form of relationship requires focus for rehabilitation, repair, or restitution of some sort. Other contexts in which moral injuries might occur—such as educational settings, among other helping professions (85), and among journalists and refugees (34), for example—are also characterized by and vulnerable to inherent structural relations that could have primary relevance for case construction.

Additionally, moral injuries might arise in contexts of dysfunction in one’s relationships in other social, communal, and religious formations such as among family members, one’s immediate local communities, or in contexts of faith communities. Moral injuries emerging from experiences of displacement (86, 87) or chronic exposure to systemic harms such as racism (45) may also be better understood through the relational framework, as these experiences involve ongoing disruptions to the relational conditions that sustain moral engagement rather than discrete event exposures.

## 7 Conclusion

Undertaking what we have described as a Copernican Revolution in the conceptual framework of moral injury research does not necessarily produce a replacement definition of moral injury. But it does lead to redirecting attention from inferred intrapsychic structures to observable relational practices and the elaborate ways these shape expectations of self and others. What the shift provides is not an answer to the question *what is a moral injury* but a more productive set of questions. Critically, these include questions that can be investigated with empirical tools clinical science already has at its disposal.

At the broadest level, the relational framework generates two overarching predictions. First, the severity of moral injury should be better predicted by the degree of perceived relational disruption—loss of belonging, rupture of trust, collapse of mutual recognition—than by abstract belief inconsistency alone. Second, if moral beliefs are understood not as free-standing cognitive structures but as representational traces of relational experience (e.g., “I am unforgivable” as an expression of ruptured self-relation; “people are unsafe” as an expression of collapsed trust), then measures of social pain and trust disruption should mediate the link between PMIE exposure and functional impairment. We argue that the proximate injury is in moral relatedness; beliefs, where they are altered, are one downstream expression.

### 7.1 Limitations and further implications

Our emphasis on the relational context as a foreground consideration in identifying how moral injuries might occur does not necessarily lead to a new definition. It is also important to note that even in the relational framework, persons’ moral beliefs are obviously relevant to their moral experiences even though we claim that it is not the beliefs or belief structures that are themselves subject to damage.

A relational model of moral injury that looks somewhat like we have presented above could reduce the number of assumptions in moral injury conceptualization, be more testable, and better account for the efficacy of current relational-focused treatment models. It could also turn out to be the case that when taken up by others with the relevant expertise that the relational model also requires assumptions that grow unwieldy when applied to particular cases. We hypothesize that the most relevant change agents with the greatest likelihood of providing “lasting corrective and humanizing pro-social experiences” (2, p. 251) will be germane to the *type of relationship* and *experiential conditions* of rupture for the person suffering MI. Identifying these could potentially lead to better outcomes for patients as well as their families, coworkers, and others in their communities. While our findings and speculations require additional development and testing to have actionable application in clinical contexts, we believe the conceptual analysis presented here serves as sufficient justification for reconsideration of the prevailing conceptual paradigm.

## Data Availability

The original contributions presented in the study are included in the article/supplementary material. Further inquiries can be directed to the corresponding author.

## Footnotes

1 For philosophers with interests in moral phenomenology, this idea is central to Immanuel Kant’s contributions to epistemology and conception of human understanding when he argued that our perception of the world is fundamentally informed by the structure of mind rather than mind conforming to the world in itself. Later phenomenologists, such as Edmund Husserl, develop analytic methods for studying the structure of consciousness that shapes human perception, interpretation, and understanding (26–28). More recently, Thomas Kuhn distinguishes what he calls “normal science” that advances progressively from breakthroughs that arise from paradigmatic transformations (29). In medicine, such revolutions occur when prevailing theoretical models are replaced by new ones, as seen in germ theory replacing miasma theory. A “Copernican Revolution” can advance scientific inquiry and clinical practice, which can lead to more accurate and effective approaches to health and disease. 

2 Our view is also amenable to non-human beings as important in the web of relations in which one participates. These might include non-human animals, spiritual entities, etc., and these relationships could be important for case construction in particular cases.

3 We are grateful to the manuscript reviewers for this and other suggestions that have strengthened the paper. We are also grateful to members of the Moral Injury Reading Group at the University of Virginia, especially Professor Jennifer Geddes, for posing challenging questions and encouragement as we developed this paper.
